# Dual‐Segment Colonic Volvulus Involving the Sigmoid and Transverse Colon: A Surgical Emergency

**DOI:** 10.1155/cris/5273726

**Published:** 2026-05-12

**Authors:** Gourav Goyal, Jyoti Meena, Mansi Singh

**Affiliations:** ^1^ Department of Surgery, People’s Medical College Hospital and Research Centre, Bhopal, Madhya Pradesh, India; ^2^ Department of Surgery, Atal Bihari Vajpayee Government Medical College, Vidisha, Madhya Pradesh, India; ^3^ Department of Medicine, Bogomolets National Medical University, Kyiv, Ukraine, nmu.edu.ua

## Abstract

Synchronous colonic volvulus is an extremely rare and life‐threatening cause of large bowel obstruction, characterized by simultaneous torsion of two separate colonic segments. Early diagnosis is challenging due to its nonspecific clinical presentation and overlapping radiological features with isolated volvulus. Delay in recognition increases the risk of ischemia, perforation, sepsis, and mortality, making rapid identification and surgical intervention critical. We present a case of a middle‐aged patient who arrived at the emergency department with progressive abdominal pain, distension, constipation, and absence of flatus. Physical examination revealed a markedly distended abdomen with diffuse tenderness, and laboratory tests demonstrated leukocytosis. Abdominal radiographs suggested large bowel obstruction, while contrast‐enhanced computed tomography (CECT) demonstrated the characteristic whirl sign at two distinct anatomical sites, consistent with synchronous volvulus of the sigmoid and transverse colon. The patient underwent emergency exploratory laparotomy, which confirmed volvulus of both affected segments with compromised vascularity. Surgical management included resection of the necrotic segments with primary anastomosis. The postoperative course was uneventful, and the patient was discharged in stable condition with scheduled follow‐up. This case highlights the importance of maintaining a high index of suspicion in patients presenting with acute large bowel obstruction, particularly when imaging demonstrates atypical or dual transition points. While sigmoid volvulus is the most common form, synchronous involvement of multiple colonic segments is exceedingly uncommon but should be considered to prevent diagnostic delay. Timely surgical intervention remains the cornerstone of management and is paramount in preventing progression to gangrene, peritonitis, and septic shock.

## 1. Introduction

Colonic volvulus is a pathological twisting of the bowel around its mesenteric axis, resulting in luminal obstruction and potential vascular compromise. It accounts for approximately 1%–10% of large bowel obstruction cases globally, with notable geographic variation influenced by diet, anatomical predisposition, and socioeconomic factors [1, 2]. The sigmoid colon is most frequently involved, responsible for nearly 75% of all cases, while volvulus of the cecum accounts for approximately 22% [3, 4]. In contrast, volvulus of the transverse colon and splenic flexure is uncommon, representing only 1%–3% of cases [5].

Synchronous volvulus involving more than one colonic segment is exceptionally rare and represents a diagnostic and therapeutic challenge due to its nonspecific clinical presentation and limited recognition in routine clinical practice [6, 7]. Typical symptoms include abdominal distension, pain, constipation, and vomiting, although these may overlap with other causes of bowel obstruction, contributing to delays in diagnosis [1, 8]. Radiological assessment plays a pivotal role in early recognition. While abdominal radiographs may demonstrate air‐fluid levels or gross distension, contrast‐enhanced computed tomography (CECT) is considered the most reliable modality due to findings such as the characteristic whirl sign and transition points [9, 10].

Management depends on bowel viability, stability of the patient, and anatomical findings. Endoscopic decompression may be attempted in isolated sigmoid volvulus but is associated with recurrence and is generally unsuitable for synchronous volvulus [11]. Surgical intervention remains the mainstay of treatment and is crucial to prevent ischemia, perforation, sepsis, and mortality [4, 12].

Given the rarity of synchronous colonic volvulus, each reported case adds valuable information to the literature and may help improve early recognition, diagnostic pathways, and management strategies.

## 2. Case Presentation

A 52‐year‐old male presented to the emergency department with progressively worsening abdominal pain and distension for 3 days, accompanied by absolute constipation, inability to pass flatus, and multiple episodes of vomiting. There was no history of fever, previous abdominal surgeries, chronic illness, or similar prior complaints. On arrival, the patient was hemodynamically stable.

On physical examination, the abdomen appeared markedly distended. Percussion revealed a tympanic note, and auscultation showed minimal and hypoactive bowel sounds. Generalized abdominal tenderness was noted, more pronounced in the epigastric region, but there was no guarding, rigidity, or rebound tenderness. No clinical signs of peritonitis were present. Digital rectal examination revealed an empty rectum without palpable masses.

**Figure 1:**
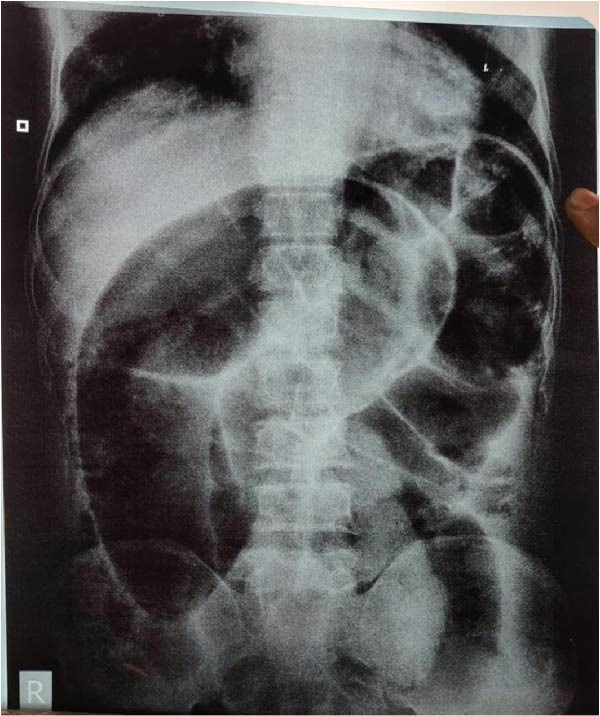
Supine abdominal X‐ray showing markedly dilated bowel loops and air‐fluid levels consistent with large bowel obstruction.

**Figure 2:**
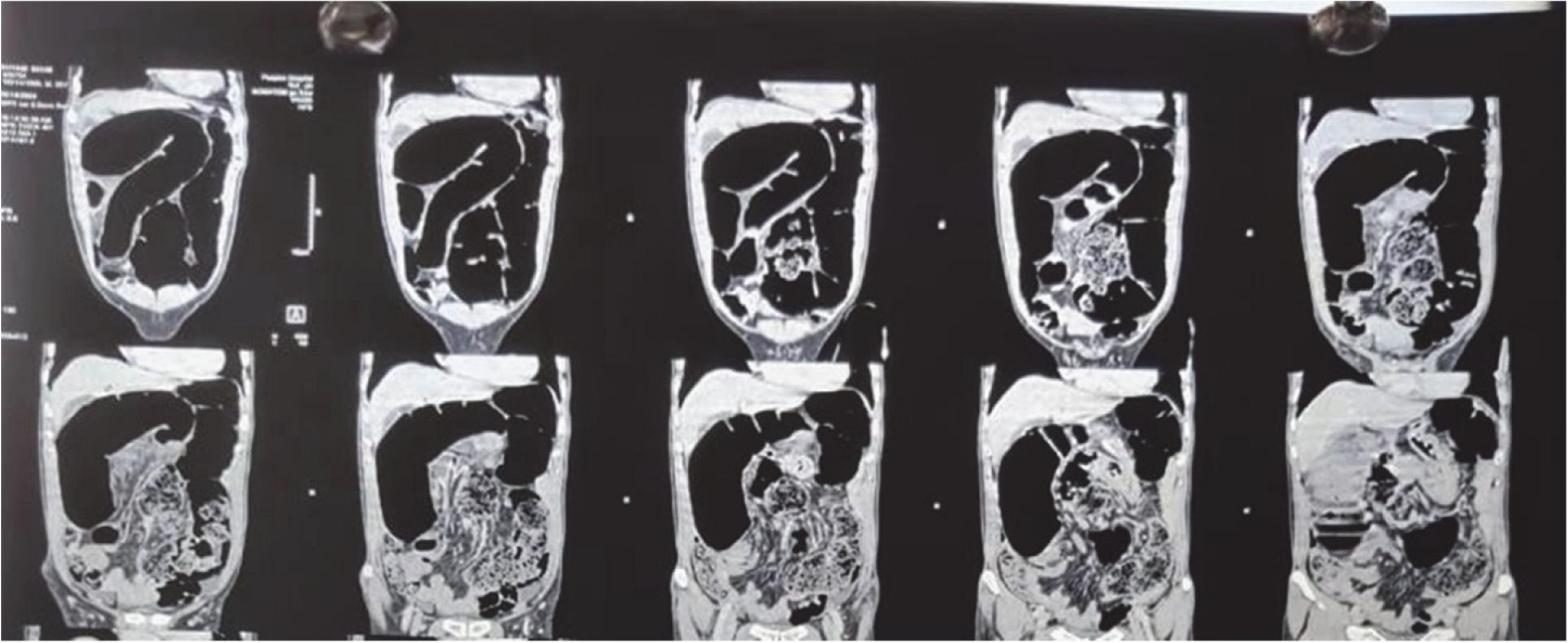
Coronal CT scan demonstrating dilated colonic segments and whirl‐like twisting of mesenteric vessels, suggestive of synchronous volvulus.

Initial imaging included an erect chest radiograph and a supine abdominal radiograph. The chest radiograph was unremarkable with no evidence of pneumoperitoneum, while the abdominal radiograph demonstrated markedly dilated bowel loops with prominent air‐fluid levels, suggestive of large bowel obstruction (Figure 1). CECT of the abdomen was performed for further evaluation and demonstrated significantly dilated colonic loops with twisting of mesenteric vessels and a characteristic whirl sign, raising suspicion for synchronous colonic volvulus (Figure 2).

Several differential diagnoses were considered in this patient presenting with acute large bowel obstruction, including:

• Isolated sigmoid volvulus: This was initially suspected given its higher prevalence. However, CECT demonstrated two distinct transition points and whirl signs, which suggested involvement of more than one colonic segment, thereby excluding isolated disease.
• Colonic malignancy causing obstruction: Large bowel obstruction due to malignancy is common in this age group. However, the absence of a focal mass lesion, irregular wall thickening, or lymphadenopathy on computed tomography (CT) made this diagnosis unlikely.
• Adhesive bowel obstruction: Although adhesions are a frequent cause of obstruction, the patient had no prior surgical history, and imaging findings were more consistent with volvulus rather than adhesive bands.
• Pseudo‐obstruction (Ogilvie’s syndrome): This was considered due to marked colonic distension; however, the presence of mechanical twisting (whirl sign) on CT ruled out functional obstruction.
• Fecal impaction or severe constipation: These conditions can mimic obstruction but were excluded based on imaging, which demonstrated true luminal obstruction with vascular compromise rather than fecal loading.

Based on the clinical presentation and characteristic radiological findings, a diagnosis of synchronous volvulus involving the sigmoid and transverse colon was established.

**Figure 3:**
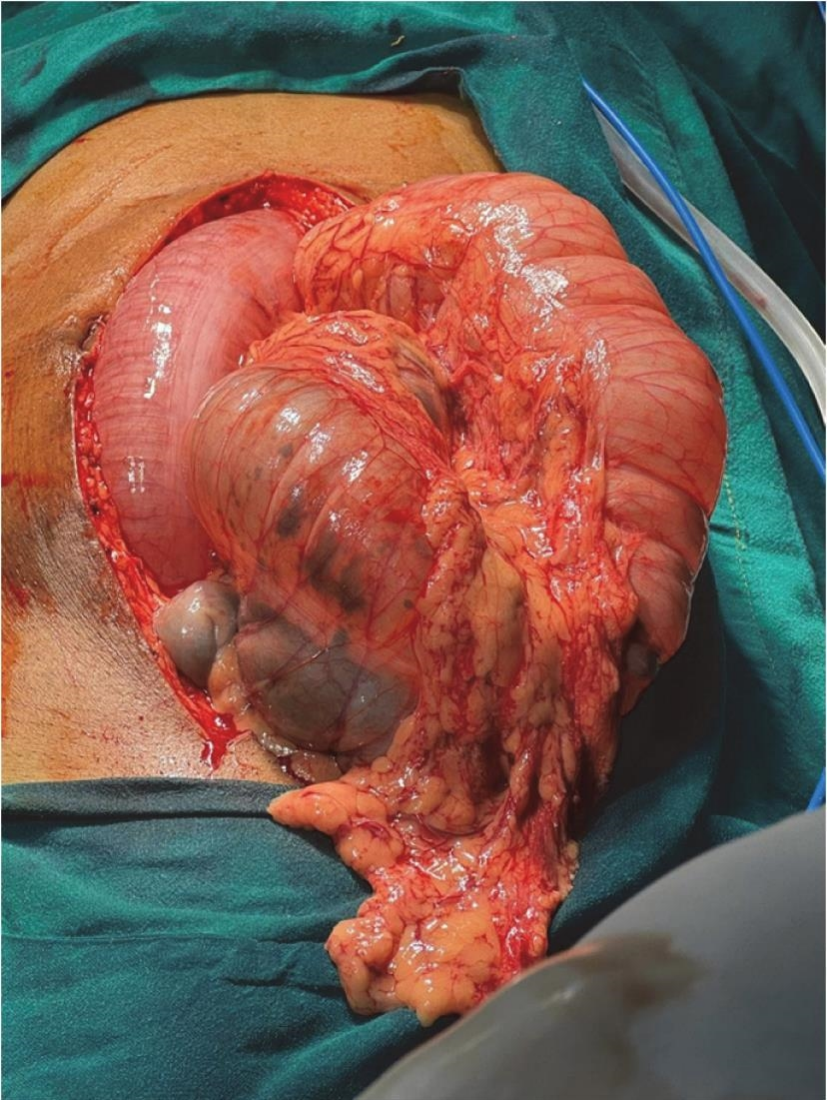
Intraoperative image showing twisted and distended colonic loops involving both transverse and sigmoid segments.

After adequate resuscitation, the patient underwent emergency exploratory laparotomy. Intraoperatively, dense adhesions were noted between the cecum, ascending colon, and transverse colon. Both transverse colon volvulus and sigmoid volvulus were identified without evidence of ischemia or gangrene (Figure 3). Controlled colonic decompression was performed through a small transverse colotomy, followed by sigmoid decompression using a flatus tube. The volvulus segments were derotated, adhesions released, and an intra‐abdominal pelvic drain was placed. The abdomen was closed in layers.

The postoperative course was uneventful. The flatus tube was removed on postoperative day 5, and the pelvic drain was removed on day 8. The patient was discharged in stable condition with advice for regular outpatient follow‐up. Follow‐up visits were scheduled at 2 weeks and 1 month postdischarge to monitor for recurrence or postoperative complications. During follow‐up, the patient remained asymptomatic, with normal bowel habits and no evidence of abdominal pain, distension, or obstruction. No postoperative complications or recurrence were noted.

## 3. Discussion

Synchronous volvulus of the sigmoid and transverse colon is a rare clinical entity, with comparatively few cases described in the literature [6, 7]. The sigmoid colon is particularly prone to volvulus due to its elongated mesentery and increased mobility, whereas volvulus of the transverse colon is rare because of stronger ligamentous fixation and anatomical stability [3, 5]. The simultaneous involvement of both segments increases the diagnostic complexity and risk of rapid progression to ischemia or perforation if treatment is delayed [4, 12].

The exact etiology of synchronous colonic volvulus remains poorly understood due to its rarity. Known predisposing factors for colonic volvulus include chronic constipation, high‐fiber diet, elongated mesentery, colonic redundancy, prior abdominal surgery, and neuropsychiatric disorders [1, 4]. However, in the present case, no clear predisposing factor was identified.

Notably, intraoperative findings demonstrated dense adhesions between adjacent colonic segments, which may have altered normal bowel mobility and contributed to torsion. Although the patient had no prior surgical history, these adhesions may represent subclinical inflammatory processes or congenital variations. Additionally, an underlying redundant colon or mesenteric elongation may have contributed to the development of synchronous volvulus.This case highlights that synchronous volvulus can occur even in the absence of classical risk factors, emphasizing the need for clinical vigilance.

Patients typically present with abdominal pain, bloating, constipation, and vomiting, although symptoms may overlap with other causes of bowel obstruction, leading to diagnostic uncertainty [1, 8]. In this case, the patient presented after 3 days of progressive symptoms, consistent with the delayed presentation frequently reported in synchronous volvulus. A range of differential diagnoses were considered, including isolated sigmoid volvulus, colonic malignancy, adhesive obstruction, and pseudo‐obstruction. However, the presence of dual whirl signs and multiple transition points on CT imaging was highly suggestive of synchronous volvulus, allowing exclusion of these alternative diagnoses.

Radiographic evaluation is central to diagnosis. Although abdominal X‐rays may show a markedly dilated bowel and air–fluid levels, CT scanning is the most sensitive and specific modality. Classic CT findings include the whirl sign, transition points, and proximal colonic dilation, which are particularly valuable in identifying synchronous disease [9, 10]. These radiologic features guided the surgical decision‐making in this case.

Management of synchronous volvulus depends largely on bowel viability and the presence of ischemia. Endoscopic detorsion is considered safe for isolated sigmoid volvulus but has limited benefit in synchronous presentations due to increased recurrence and incomplete reduction [11]. Therefore, surgical exploration remains the definitive treatment, particularly when ischemia, failed endoscopic reduction, or dual‐segment involvement is suspected [4, 12].

In this patient, timely surgical detorsion and decompression prevented ischemia and negated the need for bowel resection. The prognosis of colonic volvulus largely depends on the timeliness of intervention and bowel viability at presentation. In cases where early diagnosis and surgical management are achieved before the onset of ischemia or perforation, outcomes are generally favorable. In the present case, prompt intervention resulted in complete recovery without postoperative complications.

However, the risk of recurrence remains a concern, particularly in patients managed without definitive resection. Studies suggest that non‐resectional procedures such as detorsion alone may be associated with higher recurrence rates compared to segmental resection [10, 11]. Although no recurrence was observed during short‐term follow‐up in this patient, the possibility of future volvulus cannot be entirely excluded.

Close clinical follow‐up is therefore recommended, and patients should be counseled regarding early symptoms of recurrence. Consideration of definitive surgical intervention may be warranted in cases of recurrent volvulus.

Synchronous volvulus involving both the sigmoid and transverse colon is an exceptionally rare clinical entity, with only a limited number of cases reported in the literature [13]. Most previously reported cases present as acute large bowel obstruction, characterized by abdominal pain, distension, constipation, and vomiting—features that closely mirror the presentation observed in our patient [14]. Similar to our case, diagnosis is often challenging preoperatively and is frequently confirmed intraoperatively due to overlapping radiological features with isolated sigmoid volvulus [15].

Radiologically, prior reports emphasize the importance of CT findings such as the whirl sign and multiple transition points, which are considered highly suggestive of synchronous involvement and were also observed in our case [14]. However, in many earlier cases, the diagnosis was not established until surgical exploration, highlighting the diagnostic difficulty associated with this condition [15, 16].

In terms of management, most reported cases have required emergency surgical intervention, often involving segmental or subtotal colectomy due to bowel ischemia or necrosis [13, 17, 18]. In contrast, our patient was managed successfully with detorsion and decompression without bowel resection, as no ischemia or gangrene was identified intraoperatively. This conservative surgical approach has been less commonly reported and underscores the importance of early diagnosis and timely intervention in preserving bowel viability [19].

This case reinforces the importance of maintaining a high index of suspicion for synchronous volvulus, particularly when imaging suggests atypical twisting or multiple transition points. Reporting such cases is essential to improving clinical recognition and guiding operative decision‐making.

## 4. Conclusion

Synchronous volvulus of the sigmoid and transverse colon is an exceptionally rare presentation of large bowel obstruction and can pose significant diagnostic and therapeutic challenges. Early recognition is critical, as delayed treatment increases the risk of ischemia, perforation, sepsis, and mortality. CECT remains the cornerstone of diagnosis due to its ability to identify the whirl sign, transition points, and extent of obstruction. Surgical management remains the definitive treatment, with the approach determined by bowel viability and anatomical findings. This case reinforces the importance of maintaining clinical suspicion for synchronous volvulus when radiological or intraoperative findings are atypical and underscores the value of timely operative intervention to achieve favorable outcomes.

### 4.1. Learning Points

• Synchronous volvulus should be considered in patients presenting with severe abdominal distension and unusual imaging patterns suggestive of multiple transition points.
• CECT is the most sensitive diagnostic modality for detecting volvulus, especially when the presentation is atypical.
• Endoscopic reduction has limited value in synchronous volvulus and is generally not recommended as definitive therapy.
• Surgical intervention should be prompt to prevent ischemia and necrosis, with management tailored to bowel viability.
• Reporting rare presentations enhances clinical awareness and may improve diagnostic and operative decision‐making in similar cases.

## Author Contributions

Gourav Goyal contributed to the conception and design of the case report, clinical management of the patient, data acquisition, and critical revision of the manuscript for important intellectual content. Jyoti Meena was involved in patient care, collection and interpretation of clinical data, literature review, and manuscript drafting. Mansi Singh contributed to data analysis and interpretation, drafting of the manuscript, literature review, and final editing.

## Funding

No funding to disclose.

## Disclosure

All authors reviewed and approved the final version of the manuscript and agree to be accountable for all aspects of the work.

## Consent

Written informed consent was obtained from the patient for publication of this case report and any accompanying images. A copy of the signed consent form is available for review by the journal’s editorial office upon request.

## Conflicts of Interest

The authors declare no conflicts of interest.

## Data Availability

The data that support the findings of this study are available from the corresponding author upon reasonable request.
